# Risk factors for 30-day mortality in patients who received DeBakey type I aortic dissection repair surgery

**DOI:** 10.1186/s13019-021-01702-9

**Published:** 2021-10-30

**Authors:** Zhigang Wang, Tao Chen, Pingping Ge, Min Ge, Lichong Lu, Lifang Zhang, Dongjin Wang

**Affiliations:** 1 Department of Cardio-Thoracic Surgery, Affiliated Drum Tower Hospital, Medical School of Nanjing University, Zhongshan Road 321, Nanjing, 210008 China; 2 Department of General Practice, Nanjing First Hospital, Nanjing Medical University, Nanjing, China; 3 Department of Psychiatry, The First Affiliated Hospital, Zhengzhou University, Zhengzhou, China

**Keywords:** Aortic dissection, 30-day mortality, Risk factors

## Abstract

**Objective:**

This study aimed to identify risk factors for 30-day mortality in patients who received DeBakey type I aortic dissection (AD) repair surgery.

**Methods:**

A total of 830 consecutive patients who received acute DeBakey type I AD surgery between 2014 and 2019 were included in the study. The associations between 30-day mortality and perioperative parameters were examined in order to identify risk factors.

**Results:**

Our data suggested that the overall 30-day mortality rate of all enrolled patients was 11.7%. Unsurprisingly, non-survivors were older and more frequently accompanied with histories of cardiovascular diseases. For intraoperative parameters, the prevalence of coronary artery bypass grafting and cardiopulmonary bypass times were increased in non-survivors. In addition, acute kidney injury (AKI), dialysis, stroke, and deep sternal wound infection were more commonly seen among non-survivors. The multivariate logistic regression analysis suggested that cardiovascular disease history, preoperative D-dimer level, drainage volume 24 h after surgery, and postoperative AKI were independent risk factors for 30-day mortality after DeBakey type I aortic dissection repair surgery.

**Conclusions:**

Our study demonstrated that cardiovascular disease history, preoperative D-dimer level, drainage volume 24 h after surgery as well as postoperative AKI were risk factors for 30-day mortality after DeBakey type I aortic dissection repair surgery.

## Introduction

Acute DeBakey type I aortic dissection (AD) is a cardiovascular emergency event that often leads to poor prognosis. The lesions in type I AD extend from the ascending aorta through the transverse aortic arch and reach the descending thoracic and thoracoabdominal aorta. The high rates of morbidity and mortality rate are associated with devastating events like aortic rupture, including rupture into the pericardium, or extension of dissection into the aortic valve or branches such as coronary arteries, supra-aortic trunks, visceral arteries, or extremities [1].

Emergency surgery has been proved to be the most effective treatment once acute type I AD is diagnosed. In the past 10 years, with the advancement of surgical technology, the mortality rate during type A AD (TAAD) surgery has been greatly reduced from 23.2 to 4.9% [2–8]. However, compared to type II AD, patients diagnosed with type I AD still suffered more perioperative complications such as intestinal malperfusion, postoperative acute kidney injury (AKI) [3, 9, 10] and 30-day mortality rate. Nevertheless, the risk factors for 30-day mortality in type I AD had not been well determined. In this study, we retrospectively reviewed 830 consecutive acute type I AD patients who received TAAD surgery to explore risk factors for in-hospital death.

## Methods

### Patients

This is a single-center retrospective study approved by the Ethics committee of Nanjing University (No. BL2014004). The informed consent was waived considering the retrospective nature of this study. This study screened a total of 861 consecutive acute type I AD patients who received TAAD surgery at Nanjing Drum Tower hospital between January 2014 and December 2019. AD was defined according to the DeBakey classification by contrast-enhanced computed tomography angiography depending on anatomical location of the lesion and the extended-range. Acute AD was considered if the time from disease onset to admission was within 14 days. Patients who had type I AD longer than 14 days and who died before surgery were excluded from the study. Eventually, a total of 830 patients were included in this study. Emergency operations were performed within 48 h of symptom onset in all patients. Postoperative AKI was diagnosed according to the Kidney Disease Improving Global outcomes criteria [11]. Previous cardiovascular disease history was defined as coronary artery disease or valvular disease that required medication treatment.

### Surgical procedures

The operation procedure performed in this study was described in our previous studies [9, 12–14]. Briefly, the root procedures included direct repair or replacement as inclusion root, Bentall procedure, or David procedure. The distal arch repair consisted of hemi-arch replacement, island arch replacement, total arch replacement (TAR), triple-branched stent, and fenestrated stent depending on patient’s preoperative status, entry tear location, and aortic diameter. The indications for TAR were as follows: (1) a primary tear in transverse arch or proximal descending aorta, and (2) large involvement of the arch vessels. For TAR procedure, after the stent (Microport, Shanghai, China) which was used as the frozen elephant trunk, was inserted into the true lumen of the descending thoracic aorta, the remaining aortic arch and descending aorta were anastomosed with an artificial vessel. Aortic arch operations were performed under hypothermic circulatory arrest with selective cerebral perfusion and open distal anastomosis.

### Statistical analysis

All statistical analysis was performed using IBM SPSS Statistics software (Version 25, IBM Corp., Armonk, NY). Continuous variables were presented as mean ± standard deviation (SD) or median (quartile) and were compared using student’s *t*-test or Mann–Whitney *U*-test depended on whether or not the variables were normally distributed. Categorical variables were presented as percentages with numbers and compared with chi-square test and Fisher’s exact test as appropriate. Multivariate logistic regression analysis was applied to identify independent risk factors of 30-day mortality following stepwise enter method, including variables with *p* < 0.20 on univariate analysis. The Kaplan–Meier analysis and log-rank test were used to compare survival between groups. Receiver operating characteristic (ROC) curve analysis was used to detect the diagnostic value for D-dimer in predicting 30-day mortality. A *p*-value of < 0.05 was considered significant.

## Results

The median age of all enrolled patients was 52 years. Among all patients, 619 (74.8%) were males, and 97 (11.7%) died within 30 days after surgery. Demographic data, baseline clinical characteristics, and laboratory variables were summarized in Table 1. Our data suggested that the average age and prevalence of previous cardiovascular diseases were increased in non-survivors. In terms of laboratory parameters, serum creatine, D-dimer, and international normalized ratio level upon admission were increased while the preoperative albumin and fibrinogen levels were significantly decreased in non-survivors compared to survivors.

**Table 1:** Comparison of preoperative variables

Variables	Total (n = 830)	Survivors (n = 733)	Non-survivors (n = 97)	*P* value^a^
*Demographic data*				
Age (year)	51.5 ± 12.6	51.8 ± 12.4	58.5 ± 12.7	** < 0.001**
Male (%)	619 (74.8)	555 (75.7)	65 (68.1)	0.108
BMI (kg/m^2^)	25.8 ± 4.8	25.9 ± 4.8	25.2 ± 4.1	0.150
*Medical history*				
Hypertension (%)	608 (73.3)	535 (73.0)	73 (75.3)	0.635
Diabetes mellitus (%)	18 (2.2)	17 (2.3)	1 (1.0)	0.711
Previous cardiac surgery (%)	36 (4.3)	32 (4.4)	4 (4.1)	1.000
Previous cardiovascular disease (%)	28 (3.4)	17 (2.3)	11 (11.3)	** < 0.001**
Cerebrovascular disease (%)	30 (3.6)	25 (3.4)	5 (5.2)	0.382
Pericardial effusion (%)	135 (16.3)	120 (16.4)	15 (15.5)	0.820
*Preoperative laboratory data*				
WBC (109/L)	11.1 (8.6, 14.1)	11.0 (8.6, 13.9)	11.9 (8.0, 15.5)	0.160
sCr (μmol/L)	81.0 (61.8, 113.0)	80.0 (61.0, 108.2)	92.9 (65.3, 155.2)	**0.006**
PLT (109/L)	152.1 ± 89.7	153.4 ± 91.1	142.7 ± 78.5	0.150
ALB (g/L)	36.7 ± 4.9	37.0 ± 4.7	34.4 ± 6.1	** < 0.001**
Fibrinogen (g/L)	2.5 ± 1.4	2.6 ± 1.4	2.2 ± 1.3	**0.010**
Triglyceride (mmol/L)	1.1 (0.7, 1.5)	1.1 (0.7, 1.6)	1.0 (0.6, 1.5)	0.334
CRP (mg/dl)	22.7 (5.0, 82.8)	23.3 (4.9, 82.4)	22.3 (5.2, 90.5)	0.626
D-dimer (ng/mL)	4.7 (2.5, 9.5)	4.5 (2.3, 9.1)	6.6 (4.0, 17.0)	**0.001**
INR	1.1 (1.0, 1.2)	1.1 (1.0, 1.2)	1.2 (1.1, 1.3)	** < 0.001**

Data presented as n (%), median (IQR), or mean ± standard deviation

BMI, body mass index; WBC, white blood cell; SCr, serum creatinine; PLT, platelet; ALB, albumin; CRP, c-reactive protein; INR, international normalized ratio

Bold values indicate significance at *P* < 0.05

^a^*P* values indicate differences between survivors and deceased patients

Unsurprisingly, the operation time for cardiopulmonary bypass and aortic cross-clamp was increased in non-survivors. However, the deep hypothermic circulatory arrest time was similar between both groups. Moreover, we discovered that the surgical procedure was more frequently extended to coronary artery bypass grafting surgery in non-survivors (Table 2). Postoperatively, the incidence of dialysis, AKI, stroke, tracheostomy, and deep sternal wound infection were significantly higher among non-survivors. In addition, it was important to point out that the drainage volume 24 h after surgery was also increased in non-survivors (Table 3).

**Table 2:** Comparison of operative variables

Variables	Total (n = 830)	Survivors (n = 733)	Non-survivors (n = 97)	*P* value^a^
TAR (%)	484 (58.3)	432 (58.9)	52 (53.6)	0.317
MVR/MVP/TVP (%)	30 (3.6)	25 (3.4)	5 (5.2)	0.382
CABG (%)	49 (5.9)	34 (4.6)	15 (15.5)	** < 0.001**
Aortic valve (%)	209 (25.2)	181 (24.7)	28 (28.9)	0.374
CPB time (min)	237.2 ± 70.1	231.5 ± 64.1	283.0 ± 95.8	** < 0.001**
Aortic cross-clamp time (min)	165.7 ± 57.2	163.1 ± 53.5	186.7 ± 78.5	**0.005**
DHCA time (min)	30.7 ± 12.4	30.7 ± 12.6	30.8 ± 10.6	0.985
Intra-operative transfusion volume (ml)	325.6 ± 310.7	286.3 ± 288.7	646.8 ± 579.6	** < 0.001**

Data presented as n (%), median (IQR), or mean ± standard deviation

TAR, total arch replacement; MVR, mitral valve replacement; MVP, mitral valvuloplasty; TVP, tricuspid valvuloplasty; CABG, coronary artery bypass graft; CPB, cardiopulmonary bypass; DHCA, deep hypothermic circulatory arrest

Bold values indicate significance at *P* < 0.05

^a^*P* values indicate differences between survivors and deceased patients

**Table 3:** Comparison of postoperative variables

Variables	Total (n = 830)	Survivors (n = 733)	Non-survivors (n = 97)	*P* value^a^
Drainage volume 24 h after surgery (ml)	747.9 ± 672.3	708.4 ± 601.2	1170.9 ± 1115.1	** < 0.001**
Post-operative transfusion volume (ml)	582.6 ± 510.4	531.7 ± 484.9	922.5 ± 871.0	** < 0.001**
Re-exploration for bleeding (%)	32 (3.9)	28 (3.8)	4 (4.1)	0.782
Dialysis (%)	144 (17.3)	87 (11.9)	57 (58.8)	** < 0.001**
AKI (%)	445 (53.6)	370 (50.5)	75 (77.3)	** < 0.001**
Ventilation time (hour)	17.0 (11.5, 43.0)	16.5 (11.2, 42.0)	34.0 (20.1, 117.8)	** < 0.001**
Stroke (%)	74 (8.9)	60 (8.2)	14 (14.4)	**0.042**
Paraplegia (%)	29 (3.5)	26 (3.5)	3 (3.1)	1.000
Tracheostomy (%)	39 (4.7)	30 (4.1)	9 (9.3)	**0.037**
Deep sternal wound infection (%)	14 (1.7)	9 (1.2)	5 (5.2)	**0.017**
ICU stay time (day)	7.1 ± 10.8	7.0 ± 11.2	7.5 ± 6.1	0.507
Hospital stay time (day)	21.8 ± 12.2	23.3 ± 11.8	10.1 ± 8.3	** < 0.001**

Data presented as n (%), median (IQR), or mean ± standard deviation

AKI, acute kidney injury; ICU, intensive care unit

Bold values indicate significance at *P* < 0.05

^a^*P* values indicate differences between survivors and deceased patients

As shown in Table 4, we conducted a multivariate logistic regression analysis with above parameters and the results indicated that previous cardiovascular disease [odds ratio (OR) 53.172, 95% confidence interval (CI) 4.094–690.524; *p* = 0.002], D-dimer level upon admission (OR 1.018, 95% CI 1.000–1.035; *p* = 0.045), drainage volume 24 h after surgery (OR 1.001, 95% CI 1.000–1.001; *p* = 0.003), and postoperative AKI (OR 2.886, 95% CI 1.082–7.696; *p* = 0.034) were factors that independently associated with 30-day mortality (Hosmer–Lemeshow test *p* = 0.587). To better examine their influences on short-term outcomes, we examined the occurrence of most often and severe complications in patients with identified risk factors and presented in Table 5.

**Table 4:** Multivariate analysis of risk factors for 30-day mortality

Variable	OR	95% CI	*P* value
Age	1.022	0.988–1.056	0.210
Female	1.446	0.561–3.724	0.445
BMI	0.933	0.867–1.004	0.065
Previous cardiovascular disease	53.172	4.094–690.524	**0.002**
WBC	1.010	0.928–1.100	0.810
PLT	1.004	0.996–1.011	0.349
sCr	1.000	0.997–1.003	0.994
Fibrinogen	0.869	0.587–1.288	0.485
ALB	0.950	0.875–1.032	0.225
D-dimer	1.018	1.000–1.035	**0.045**
INR	0.824	0.296–2.291	0.710
CPB time	1.003	0.993–1.013	0.519
Aortic cross-clamp time	1.003	0.992–1.014	0.583
Concomitant CABG	0.836	0.173–4.045	0.824
Drainage volume 24 h after surgery	1.001	1.000–1.001	**0.003**
Postoperative AKI	2.886	1.082–7.696	**0.034**

OR, odds ratio; CI, confidence interval; BMI, body mass index; WBC, white blood cell; PLT, platelet; SCr, serum creatinine; ALB, albumin; INR, international normalized ratio; CPB, cardiopulmonary bypass; CABG, coronary artery bypass graft; AKI, acute kidney injury, CRP, c-reactive protein; INR, international normalized ratio

Bold values indicate significance at *P* < 0.05

**Table 5:** Comparisons of early outcomes in patients with risk factors for 30-day mortality

Variables	D-dimer (value ≥ 3.91 ng/mL versus value < 3.91 ng/mL; *P* value)	Drainage volume 24 h after surgery (value ≥ 1000 mL versus value < 1000 mL; *P* value)	Previous cardiovascular disease (Previous cardiovascular disease versus the rest; *P* value)	AKI (AKI versus non-AKI; *P* value)
Drainage volume 24 h after surgery (ml)	520.0 (300.0, 912.5) versus 500.0 (300.0, 815.0); *P* = 0.065	–	560.0 (377.5, 1392.5) versus 510.0 (300.0, 1378.0); *P* = **0.001**	540.0 (320.0, 860.0) versus 500.0 (300.0, 820.0); *P* = **0.013**
Re-exploration for bleeding	5.3% versus 3.6%; *P* = 0.229	8.3% versus 3.1%; *P* = **0.002**	14.3% versus 4.0%; *P* = **0.009**	4.7% versus 3.9%; *P* = 0.562
Dialysis	22.1% versus 12.7%; *P* < **0.001**	28.0% versus 13.7%; *P* < **0.001**	35.7% vs. 16.3%; *P* = **0.007**	–
AKI	58.4% vs. 49.6%; *P* = **0.011**	60.1% vs. 51.6%; *P* = **0.039**	71.4% vs. 53.0%; *P* = 0.054	–
Ventilation time (hour)	18.0 (12.0, 45.6) vs. 17.0 (11.3, 42.3); *P* = 0.942	24.0 (14.0, 86.5) versus 16.5 (10.5, 39.0); *P* < **0.001**	14.3 (12.0, 45.6) versus 17.0 (11.3, 42.3); *P* = 0.367	25.5 (14.0, 67.0) versus 14.5 (10.0, 21.5); *P* < **0.001**
Stroke	8.7% versus 8.2%; *P* = 0.811	11.4% versus 7.5%; *P* = 0.091	3.6% versus 8.6%; *P* = 0.346	8.1% versus 8.8%; *P* = 0.702
Paraplegia	3.7% versus 3.1%; *P* = 0.649	6.2% versus 2.5%; *P* = **0.012**	3.6% versus 3.4%; *P* = 0.953	4.5% versus 2.1%; *P* = 0.054
Tracheostomy	5.5% versus 3.8%; *P* = 0.230	4.1% versus 4.7%; *P* = 0.742	0 versus 4.7%; *P* = 0.634	6.5% versus 2.3%; *P* = **0.004**
Deep sternal wound infection	2.4% versus 0.9%; *P* = 0.087	1.0% versus 1.7%; *P* = 0.498	0 versus 1.6%; *P* = 1.000	1.6% versus 1.6%; *P* = 0.987
ICU stay time (day)	4.0 (3.0, 7.0) versus 4.0 (3.0, 6.0); *P* = 0.466	6.0 (3.0, 8.0) versus 4.0 (3.0, 6.0); *P* < **0.001**	4.0 (3.0, 5.0) versus 4.0 (3.0, 6.0); *P* = 0.966	5.0 (3.0, 8.0) versus 3.0 (3.0, 5.0); *P* < **0.001**
Hospital stay time (day)	19.0 (14.0, 25.0) versus 20.0 (15.0, 26.0); *P* = **0.025**	23.0 ± 15.0 versus 21.4 ± 11.9; *P* = 0.103	24.0 (17.5, 25.8) versus 19.0 (14.0, 25.0); *P* = 0.320	21.0 (16.0, 27.0) versus 17.0 (14.0, 23.0); *P* < **0.001**
30-day mortality	19.7% versus 4.9%; *P* < **0.001**	23.3% versus 8.2%; *P* < **0.001**	39.3% versus 10.7%; *P* < **0.001**	16.9% versus 5.7%; *P* < **0.001**

Data presented as n (%), median (IQR), or mean ± standard deviation

AKI, acute kidney injury; ICU, intensive care unit

Bold values indicate significance at *P* < 0.05

**Fig. 1:**
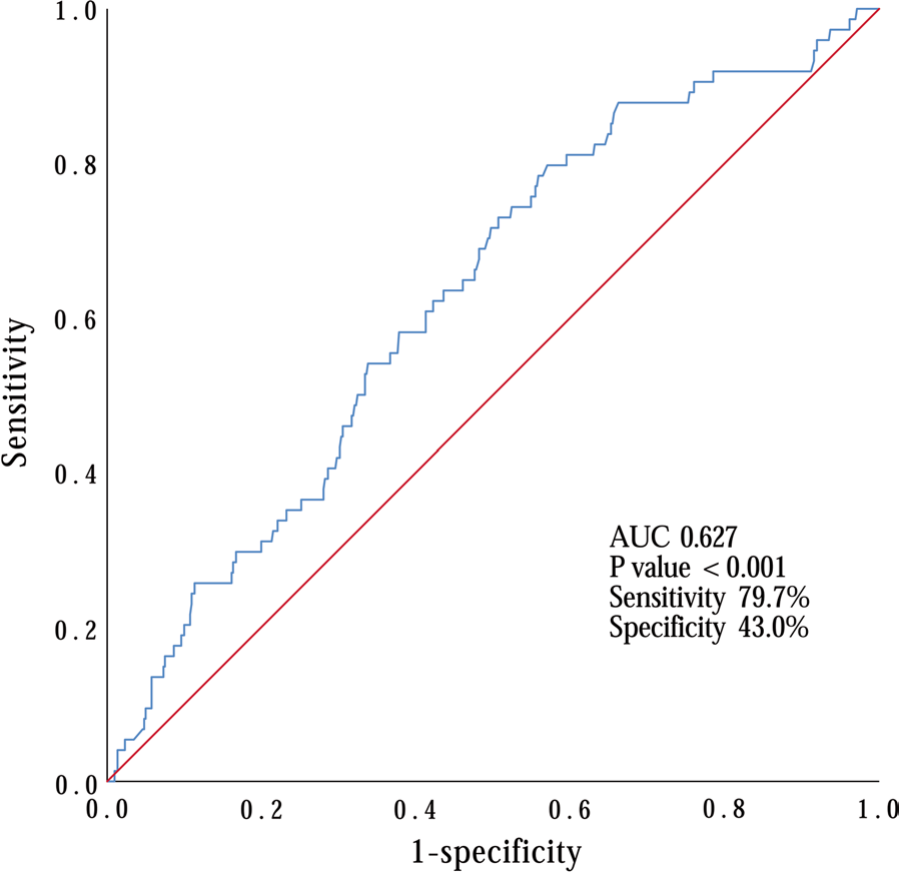
Receiver operating characteristics (ROC) curve for determination of the cut-off for prognostic D-dimer value upon admission in predicting 30-day mortality in DeBakey type I aortic dissection

**Fig. 2:**
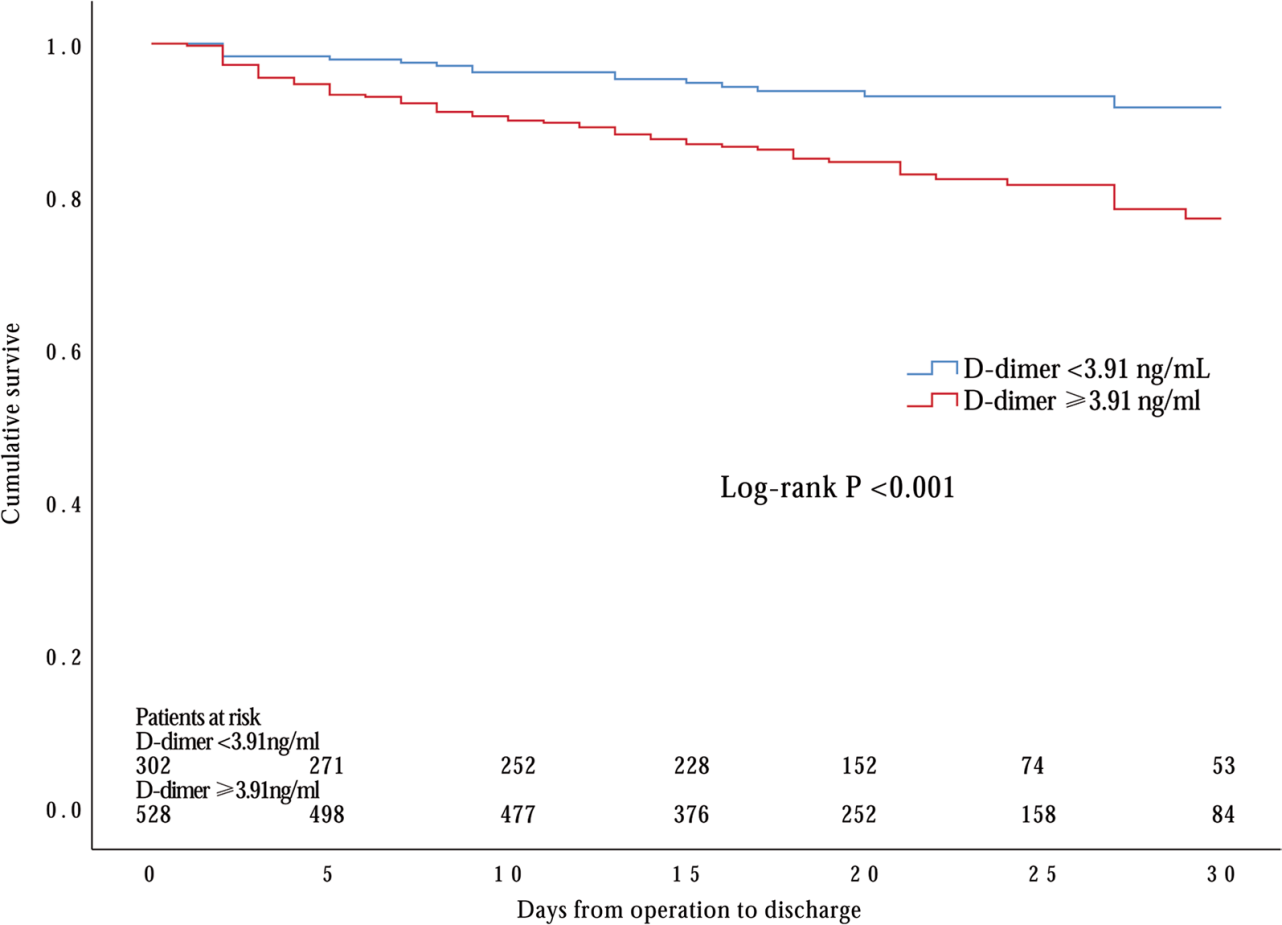
Kaplan–Meier survival curve of serum D-dimer levels upon admission in all patients with acute DeBakey type I aortic dissection treated with surgical repair

**Fig. 3:**
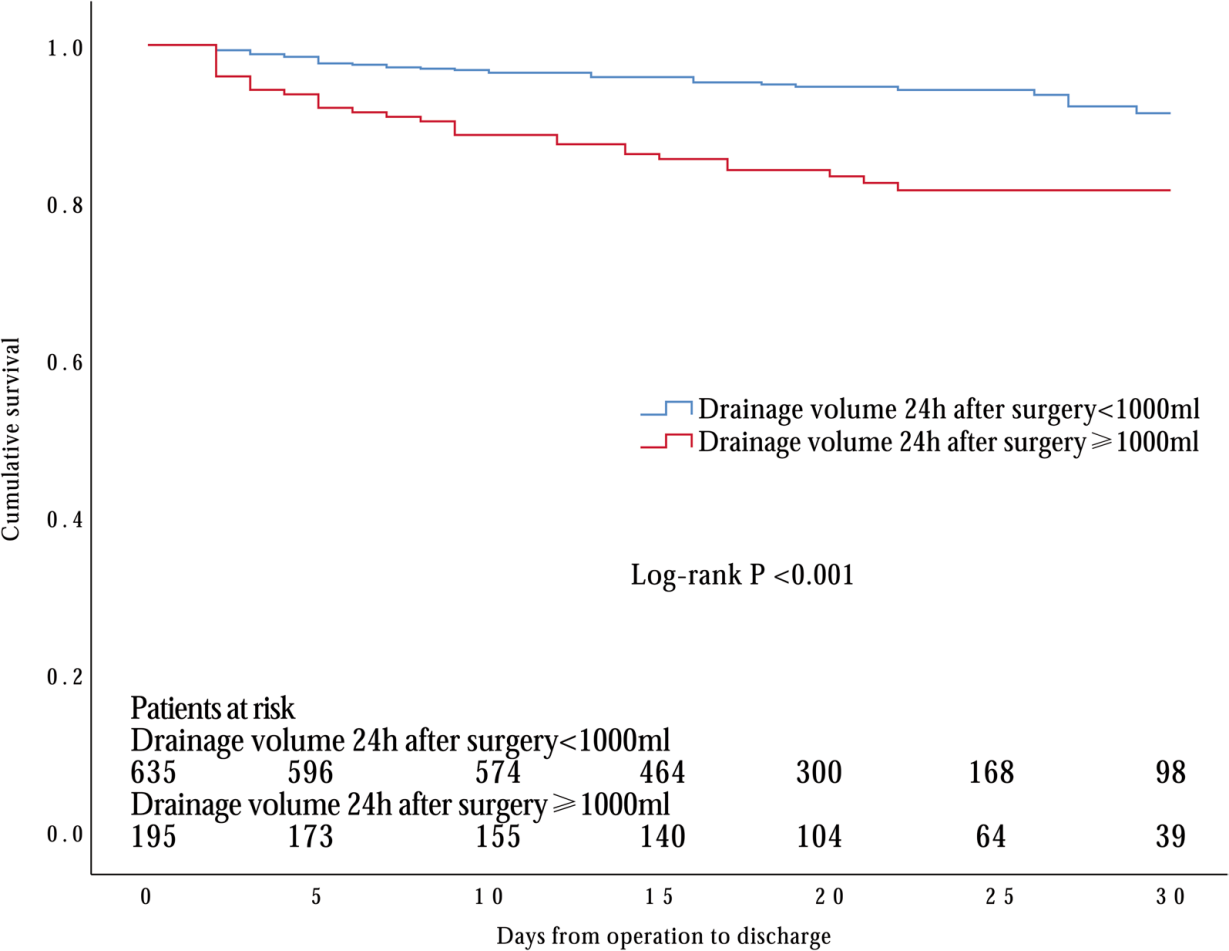
Kaplan–Meier survival curve of levels of drainage volume in all patients with acute DeBakey type I aortic dissection treated with surgical repair

Next, we examined the predictive value of D-dimer level with ROC curve analysis and revealed a significant link to 30-day death [AUC 0.627 (95% CI 0.562–0.693), *p* < 0.001] (Fig. 1). Our data suggested that values of D-dimer = 3.91 ng/mL had 79.7% sensitivity and 43.0% specificity in predicting 30-day mortality. To better confirm the results, we obtained from the multivariate logistic regression analysis. We applied Kaplan–Meier survival analysis which consistently showed that survival was significantly lower in patients with -dimer level ≥ 3.91 ng/mL upon admission as well as drainage volume ≥ 1000 ml 24 h after surgery (Log-rank *p* < 0.001 and *p* < 0.001; respectively) (Figs. 2, 3).

## Discussion

To the best of our knowledge, this was the first large cohort study evaluating risk factors for 30-day mortality in DeBakey type I AD patients who received surgical repair. Our study identified four independent factors that were associated with 30-day death after the surgery including two preoperative parameters (cardiovascular disease history, D-dimer level upon admission) and two postoperative parameters (postoperative AKI, drainage volume 24 h after surgery).

In our study, the overall 30-day mortality rate was 11.7%, which was consistent with recent similar studies of patients who received TAAD surgical repair therapy [2, 3, 8]. Considering type I AD is one of the most lethal diseases that requires instant complicated surgery, the mortality rate of our center was acceptable. Our data suggested that patients with underlying cardiovascular disease had a greater risk of postoperative death than those without cardiovascular disease histories. Myocardial ischemia is commonly seen in cardiovascular diseases. Preoperative myocardial ischemia or ST-T elevation on electrocardiogram before the operation was identified as an independent factor for increased operative mortality in TAAD patients [15–17]. Additionally, most type I AD patients with underlying cardiovascular diseases were using antiplatelet therapies such as aspirin [18], that was associated with an increased rate of postoperative major bleeding [19]. However, the concomitant coronary artery bypass grafting procedure was not identified as a risk factor, which could be explained that the coronary malperfusion problem was resolved during the operation. Therefore, our data suggested that extra attention should be paid to patients with cardiovascular disease histories.

D-dimer, a degradation product of cross-linked fibrin, was raised in all cases of TAAD and, suggesting that a normal D-dimer level can help to rule out the disease [20]. Recently, numerous studies have demonstrated that the D-dimer levels was negatively associated with short-time prognosis [21, 22]. Our study indicated that the predictive value of D-dimer was independently associated with 30-day death after surgical intervention in patients with type I AD. Contrary to our results, Li and colleagues [23] found that D-dimer level did not correlate with the risk of 30-day death after adjusting surgical intervention in TAAD patients and they proposed that the D-dimer level lost its prognostic value for 30-day death after patients received surgery. This could be explained as absolute D-dimer concentrations may reflect the anatomical extent of the disease [20]. Type I AD is a more severe and extensive type of TAAD and accompanied with permanent distal patent false lumen after surgical repair. The unique presentation and disease characteristics of Type I AD might help to explain why D-dimer levels was proved to be an independent factor for 30-day death even after adjusting for surgery in our study.

Postoperative AKI is a common serious complication in patients who received surgical repair of TAAD with a reported incidence more than 40% [24]. Our previous study identified that postoperative AKI was more frequently seen in patients with type I AD compared to patients with type II AD [9]. This might be explained by that in type I AD, the extension of dissected membrane could reach the renal vessels and results in organ malperfusion which further increases the incidence of AKI. Previous studies have indicated that postoperative AKI might increase the 30-day mortality after surgical repair of TAAD [24–26]. Consistent with our study, Fann and colleagues found that renal dysfunction per se was an independent predictor of 30-day death after surgery [27]. The mechanism underlying the postoperative AKI associated 30-day mortality might be related to postoperative AKI-induced chronic disease and end-stage renal dysfunction [28]. Olsson and colleagues had demonstrated in their study that organ failure was strongly associated with 30-day mortality in patients who received surgical repair of TAAD [29]. Thus, effective strategies are urgently required to prevent and treat postoperative AKI and thereby improve patient outcomes.

Our data revealed that drainage volume 24 h after surgery was a predictor of 30-day death. Postoperative bleeding is a leading cause of perioperative morbidity and mortality [30–32], as it could lead to hemodynamic instability or organ dysfunction in patients who received TAAD surgeries. Meanwhile, increased bleeding after surgery requires more blood transfusions. In recent years, many studies showed that postoperative blood transfusion was not only related to increasing perioperative complications, but also negatively affected the patient’s short-term survival [33, 34]. Our data suggested that an effective hemostasis strategy during operation for type I AD patients is critical in reducing 30-day mortality.

### Limitations

This study has several limitations. Firstly, although the sample size was comparably large, the cohort was collected in a single center which might representable for general population. Secondly, the difference between different surgeons and qualities of surgeries had not been evaluated in this study which might be a confounding factor for the result. Thirdly, the malperfusion related data, which has been known to be able to affect outcomes after TAAD operation [8, 35] was missing in the current dataset. Lastly, the absolute number of deaths and other major adverse events were relatively small, which might likely to reduce the statistical power for risk factor analysis. Therefore, further prospective multicenter studies are needed to assess the risk factors for in-hospital mortality and establish the most effective strategies to improve patient outcomes.

## Conclusions

Our study suggested DeBakey type I AD was a lethal disease even after emergency surgical repair with high 30-day mortality rate. The cardiovascular disease history, preoperative D-dimer level, drainage volume 24 h after surgery, as well as postoperative AKI were proved to be independent risk factors for 30-day mortality.

## Data Availability

The datasets used or analyzed during the current study are available from the corresponding author on reasonable request.

## Abbreviations

AD: Aortic dissection
TAAD: Type A AD
AKI: Acute kidney injury
TAR: Total arch replacement
ROC: Receiver operating characteristic
OR: Odds ratio
CI: Confidence interval
